# The Use of Deconstructed Tires as Elastic Elements in Railway Tracks

**DOI:** 10.3390/ma7085903

**Published:** 2014-08-18

**Authors:** Miguel Sol-Sánchez, Fernando Moreno-Navarro, Mª Carmen Rubio-Gámez

**Affiliations:** Laboratory of Construction Engineering, University of Granada, C/Severo Ochoa s/n, Granada 18071, Spain; E-Mails: msol@ugr.es (M.S.-S.); fmoreno@ugr.es (F.M.-N.)

**Keywords:** railway track, elastic elements, deconstructed tire layers, laboratory study

## Abstract

Elastic elements such as rail pads, under sleeper pads and under ballast mats are railway components that allow for a reduction in track deterioration and vibrations. And they are furthermore commonly used to obtain an optimal vertical stiffness of the infrastructure. However, the use of elastomeric materials can increase construction costs and the consumption of raw materials. Thus, the utilization of used tire layers offers an alternative to reuse an abundant waste reducing the cost of elastic elements. In addition, an innovator technique allows deconstructing tire layers without grinding up the material, reducing production costs at the same time that tire properties are remained. This research is focused on the study of the viability of developing elastic components from used tire layers by evaluating the influence of thickness, the resistance capacity of the elements and their behavior in a ballast box. Results indicate the ability of tire pads to manufacture elastic elements (rail pads, under sleeper pads and under ballast mats) to be used in railway tracks.

## 1. Introduction

Elastic elements are resilient components used in railway tracks with the aim of providing elasticity in the vertical sense, particularly in the cross-sections of tracks. Thus, these materials allow for the obtainment of an optimal vertical design of railway tracks since elastic components can be manufactured with different stiffness levels.

There are diverse types of elastic components used in railway infrastructure, but the being rail pads, under-sleeper pads, and under-ballast mats are the most spread because of its capacity to distribute loads, mitigate impacts and reduce vibrations stemming from rails, sleepers and ballast [1,2,3]. The benefits of elastic elements are associated with their polymeric nature, which provides them with both appropriate static and dynamic stiffness coefficients (kN/mm) to modify track performance and reduce stress from trains. However, the use of elastic components could lead to an important increase in track construction costs as a consequence of the price of these materials with high quality properties [4]. This fact, in combination with the implementation of sustainable policies in the railway system, has encouraged researchers, industrial manufactures and railway authorities to develop and implement elastic materials from by-industrial products.

In this way, diverse waste materials like plastics have been used in manufacturing elastic components for railway infrastructures [5,6], although one of the most successful practices is the reutilization of tire rubber to elaborate elastic elements [7,8]. Tyre rubber has high elastic properties and long-term resistance, which makes end-of-life tires appropriate for their use in railway tracks. The most extended technique consists of grinding up the rubber of the tires and mixing it with a resin binding agent. This process provokes an increase in production costs as well as the loss of mechanical properties of the tires. In this way, a recent technology allow for the obtainment of the elastic layers that form tires without grinding the material by deconstructing the end-of-life tires in the same way that they were manufactured [9]. This means that tire properties (tensile strength, thermostable properties, ageing resistance, *etc.*) remain and production costs are reduced, and at the same time an abundant waste is reused, minimizing landfill disposal.

Thus, this paper presents a research focused on the study of the viability of developing elastic components (rail pads, under-sleeper pads and ballast mats) for railway tracks from the outer layer (tread layer) of deconstructed end-of-life tires (without grinding up the material). For this purpose, the influence of tire layers thickness in each elastic element performance, the resistance to mechanical fatigue and climatic deterioration as well as the effect of tire elements in a ballast box was analyzed.

## 2. Methodology

### 2.1. Materials

**Figure 1:**
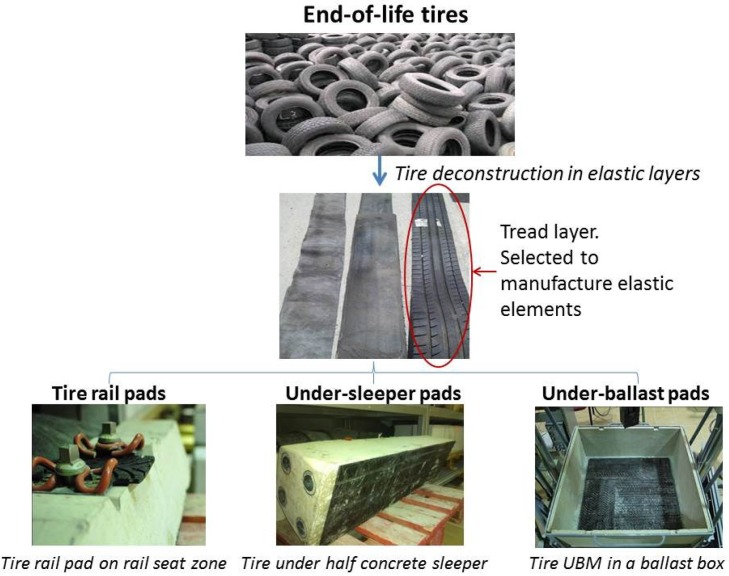
Manufacturing process of different tire elements for railway tracks.

End-of-life tires are composed of three layers: tread layer (outer), middle and inner layer (Figure 1). In this study, the outer layer was the one used to develop elastic elements with potential for being employed in railway tracks. The tread layers obtained by the deconstruction process were selected as the most appropriate material (rather than the inner and middle tire layers) since they have no metallic elements at the same time that their thickness values make them suitable to obtain elastic components with diverse vertical response. Table 1 shows the mean values of the main characteristics of outer tire layers.

**Table 1:** Tread layer properties.

Properties	Outer tire layer
Layer length (m)	1.75–1.90
Layer width (m)	0.15–0.16
Shore hardness-ISO 868	60–75
Density (g/cm^3^)-ISO 1183	1.198
Tensile strength-ISO 37 (MPa)	9.5
Elongation-ISO 37 (%)	145.0
Electrical insulation (dry)-EN 21303 (Ω)	1.24 × 10^10^
Electrical insulation (wet)-EN 21303 (Ω)	5.48 × 10^6^
Impact attenuation-EN 13146-3 (%)	>52

From outer tire layers, the following elastic elements were manufactured. Figure 1 represents a scheme of the tire elements production process.

- Rail pads with horizontal dimensions equal to 180 mm × 140 mm, which make them apt to be used under rail type UIC-54. In this study, more than 100 tire pads with thickness among 4.0 and 11.5 mm were analyzed. In addition, commercial pads (static and dynamic vertical stiffness roughly 100 kN/mm and 220 kN/mm, respectively) were employed as a reference in order to evaluate the effect of tire pads.
- Under sleeper pads (USP), which were embedded in concrete blocks with square geometry (300 mm × 300 mm). These samples are suitable for laboratory study, according to the Standard DIN 45673-6. A number of 23 elastic samples with thickness between 5.0 and 11.0 mm were used. Besides, a tire USP was embedded at the bottom surface of half concrete sleeper with the aim of studying the influence of this elastic element in ballasted tracks. Although the thickness of standard USP ranges among 5–20 mm [10,11], for this study these values were lower since rubber layers from used tires have a limited thickness.
- Under ballast mats (UBM). Elastic samples of 300 mm × 300 mm were obtained to carry out laboratory studies in reference to DBS 918 071-01. A total of 39 square samples with thickness between 11.0 and 44.0 mm were used. As the outer layer are usually thinner than 11.5 mm, diverse rubber layers were joined by a process of Temperature-Pressure-time (TPt, consisting of 2 h at 200 °C, applying 10 kg/cm^2^ of pressure) with the aim of obtaining greater thickness values. In addition, a sample with horizontal dimensions equal to 1000 × 1000 mm was used in a ballast box, being able to study the effect of tire mats in ballast box behavior.

### 2.2. Tests

With the aim of analyzing the viability of using tire elements as elastic components in railway tracks, the testing plan consists of three stages: (a) analysis of the effect of the tire layer thickness on their mechanical behavior; (b) evaluation of tire elements resistance capacity to be used in railway tracks; (c) study of tire elements in a ballast box. Table 2 summarizes the planning test developed according to European Standards to evaluate in laboratory the mechanical performance of elastic elements.

**Table 2:** Test planning developed

Study step	Elastic element	Parameter studied	Samples	Tests
influence of tire layer thickness	*rail pads*	rail pad thickness	104	-static (20–95 kN and 100–200 kN) -dynamic (20–95 kN at 4 Hz)
	*under sleeper pads*	USP thickness	23	-static (0.01–0.10 and 0.01–0.20 N/mm^2^) -dynamic 0.01–0.10 N/mm^2^ at 5–10 Hz
	*under ballast mats*	UBM thickness	39	-static (0.02–0.10 N/mm^2^) -dynamic (0.02–0.10 N/mm^2^ at 5–10 Hz)
resistance capacity	*rail pads*	mechanical fatigue	1	-fatigue over inclined sleeper (included fastener) 3 × 10^6^ load cycles -statics and dynamics
		climatic deterioration	9	-static (20–95 kN and 100–200 kN) -dynamic (20–95 kN at 4 Hz)
	*under sleeper pads*	mechanical fatigue	1	-fatigue process over ballast box (1 × 10^6^ cycles) -statics and dynamics
		climatic deterioration	9	-tensile strength
	*under ballast mats*	climatic deterioration	9	-static (0.02–0.10 N/mm^2^) -dynamic (0.02–0.10 N/mm^2^ at 5–10 Hz)
ballast box study	*rail pads*	ballast box behavior	4	-dynamics over a ballast box incorporating different elastic solutions
	*under sleeper pads*		4	
	*under ballast mats*		4	

In order to analyze the influence of the thickness of tire elastic elements, the static (at different load levels) and dynamic (at diverse load frequencies) response of rail pads (UNE-EN 13146-9 [12] and UNE-EN 13481-2 Annex B [13]), under-sleeper pads (DIN 45673-6 [14]) and under-ballast mats (DBS 918 071-01 [15]) was studied. For rail pad tests, the preload was equal to 20 kN (equivalent to the mean preload of some fastening systems [16]) whereas the maximum load was 95 kN, which correspond to 19-ton axle static load (if quasi-static forces are included, this value is reduced depending mainly on track quality and train speed [17]). Regarding load conditions for USP and UBM tests, the maximum stress was equal to 0.25 N/mm^3^ and 0.10 N/mm^3^ respectively. The frequency values were 4 Hz for rail pads, and 5 Hz and 10 Hz for USP and UBM. All these loading conditions are in consonance with those listed in European Standards for the elastic elements [12,13,14,15], being appropriate to prove the viability of using tire elements in railway tracks. However, to study their effect in high speed lines other load frequencies could be necessary depending on the track characteristics [18] as well as a study in more detail about the dynamic behavior of the elastic elements could be necessary by using other loading conditions [19,20].

On the other hand, the evaluation of the tire elements resistance was carried out by measuring the materials fatigue strength and climatic deterioration resistance. This study was developed in consonance to UNE-EN 13146-4 [21] (for rail pads), DIN 45673-6 (for USP), DBS 918 071-01 and DIN 45673-5 [22] (for UBM). In addition, static and dynamic tests were performed after fatigue and climatic processes in order to evaluate material durability.

**Figure 2:**
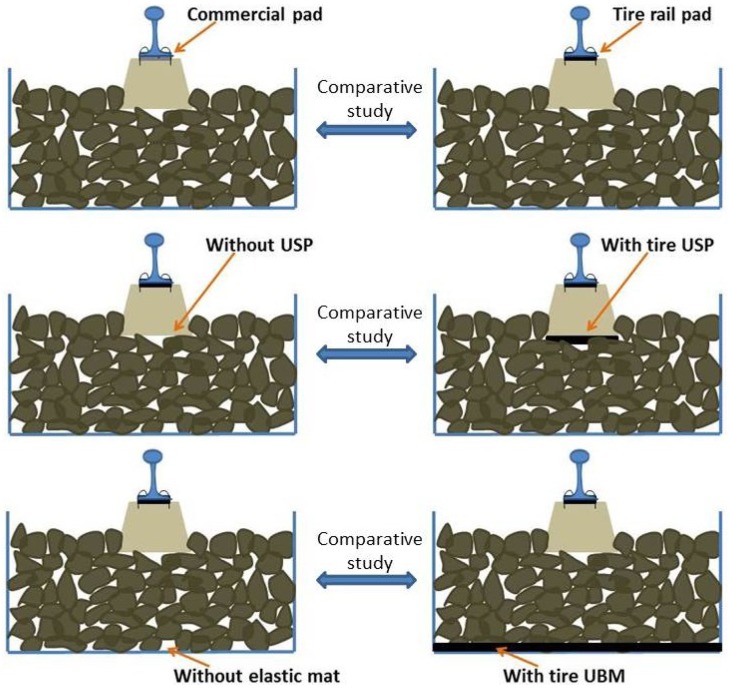
Comparative study of the effect of different elastic solutions.

Finally, the effect of tire elements was evaluated in laboratory by using a ballast box (which include sleeper, fastener and rail) considering different elastic solutions (Figure 2): (a) comparison between the system with a commercial rail pad and with a tire pad; (b) analysis of the effect of incorporating tire elements under a half concrete sleeper; (c) study of the behavior of the ballast box when a tire mat is disposed under the ballast layer, comparing the results with those recorded for the system without elastic mat. For this purpose, dynamic tests were carried out on a ballast box, applying 200,000 cycles of load between 5 kN and 65 kN at 4 Hz. The number of cycles is said to be adequate to obtain a stable behavior of the ballast layer [23], which is appropriate to achieve the objective of this study (compare the different elastic solutions).

## 3. Results and Discussion

### 3.1. Influence of Tire Thickness in Tire Pads Performance

#### 3.1.1. Rail Pads

**Figure 3:**
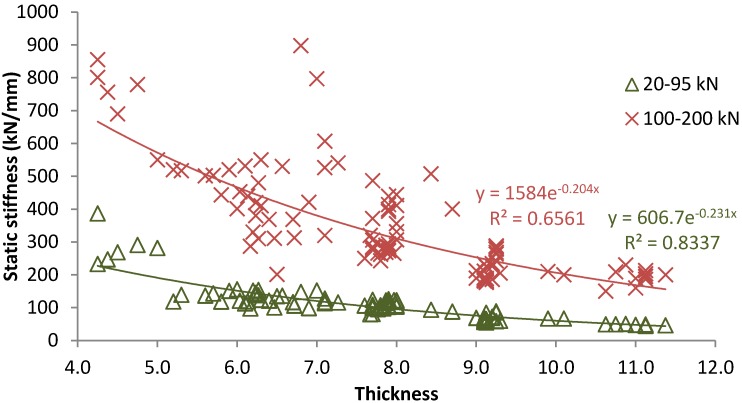
Effect of tire pads thickness on their static response.

Figure 3 shows the static stiffness measured between 20–95 kN (habitual loads in railway tracks) and 100–200 kN (high loads caused by the impact between rail and wheels due to defects in these components) for tire rail pads with thickness values among 4.0 mm and 11.5 mm. Results indicate that lower stiffness is obtained in both tests when the tire pad thickness is increased, fitting values to an exponential law. This means that a wide range of elastic solutions can be achieved by varying this design factor. In addition, in consonance with other authors [24], an increase of load level leads to the stiffening of the rubber materials as well as higher variability of results is obtained in reference to thickness values (lower R^2^).

According to [25,26], tire pads with thickness between 7.5 and 9.0 mm could be appropriate for their use as soft rail pads in high speed railway since the static stiffness is near 80–125 kN, which is the most adequate to avoid dynamic overloads by the passage of trains at the same time that the rolling resistance of trains is limited. On the other hand, tire pads with thickness lower than 7.5 mm could be appropriate for railway tracks that require stiffer pads and lower vertical deflections, obtaining higher bending capacity. Nonetheless, it is important to remark that tire pads thickness should be appropriate for practical applications. In this way, according to the literature, some thickness values used for standard pads are: 4.5, 5.0, 5.5, 6.5, 7.0, 8.0 and 10.0 [25,27].

**Figure 4:**
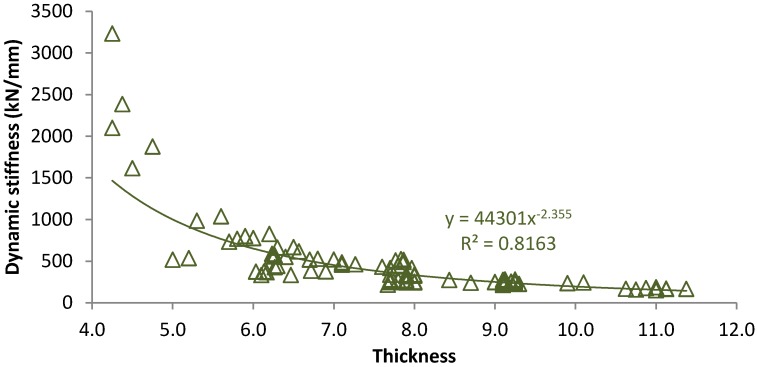
Dynamic stiffness for tire pads with diverse thickness values.

Regarding the effect of thickness on the dynamic response of tire pads, Figure 4 shows the stiffness recorded at 4 Hz for pads with thickness among 4.0 and 11.5 mm. As it can be seen, results fit to a potential law which means that material stiffness is reduced when thickness is increased, being this fact more important for thickness values lower than 6.0 mm. Besides, dynamic stiffness values are higher than those measured in static tests, which shows the material dynamic stiffening. Nonetheless, the ratio between dynamic and static results is close to 3.5 for tire pads with thickness values 6.0–9.0 mm, which is appropriate to obtain soft pads with capacity to damp loads and vibrations caused by the passage of trains [28].

In comparison with existing available rail pads, tire pads with thickness close to 5.5 mm recorded quite similar dynamic stiffness values (roughly 750 kN/mm) than those measured for commercial HDPE pads (700–900 kN/mm) [16]. Besides, tire pads present lower stiffness values than those recorded for standard rail pads made of polyurethane cork rubber (widely used in Holland) for different thickness values [27]. However, the results obtained for pads with 7.0 mm are higher than those measured for pads with similar thickness and manufactured from polyether thermoplastic elastomer (used in some Spanish High Speed Lines). In the same way, 10.0 mm thick tire pads have high stiffness values than standard studded rubber pads (about 65 kN/mm).

#### 3.1.2. Under Sleeper Pads

**Figure 5:**
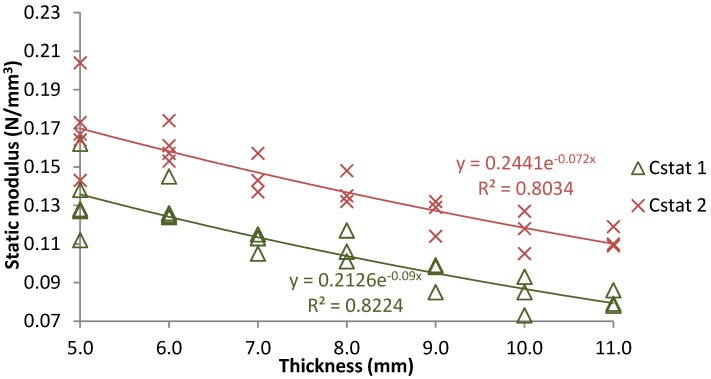
Influence of thickness in tire under sleeper pads (USP) static behavior.

Figure 5 displays the influence of thickness in the static (at different stress ranges; C_stat1_ 0.01–0.10 N/mm^2^; C_stat2_ 0.01–0.20 N/mm^2^) response of under sleeper pads manufactured from used tire layers. As it was seen for rail pads, the increase of tire thickness leads to lower stiffness modulus of USP fitting to an exponential law with R^2^ values that indicate good relationship between material stiffness and the thickness of the element. Thus, this parameter is an appropriate factor to obtain an optimal vertical design of railway tracks since it allows modifying global track vertical stiffness. In addition, it is proved that stress level is an important factor in the study of tire USP since its increase lead to higher stiffness values.

According to other authors [29], all tires USP studied in this work present appropriate thickness values to be used in railway tracks (although standard USP present thickness values up to 20 mm [10,11]) and could be classified as soft for ballast press close to 0.10 N/mm^2^. This means that this type of USP could reduce stress and vibrations transmitted by the trains to the ballast layer. On the other hand, for high ballast press (0.20 N/mm^2^), tire USP with thickness lower than 7.0 mm would allow reducing the corrugation phenomenon and the variations in stiffness transition sections as well as the ballast thickness could be reduced in critical zones such as an existing tunnel, bridge or underpass.

In comparison with the results obtained by other authors [30] who have used the same standard to develop the tests, the tire USP with 10 mm of thickness presented static stiffness values quite similar to those recorded for 4 type of USP (of 5 samples studied) with the same thickness, which have been used in European lines. Nonetheless, these laboratory results could vary depending on the type of ballast plate (metallic plate with a surface simulating ballast layer) used during the tests [31].

**Figure 6:**
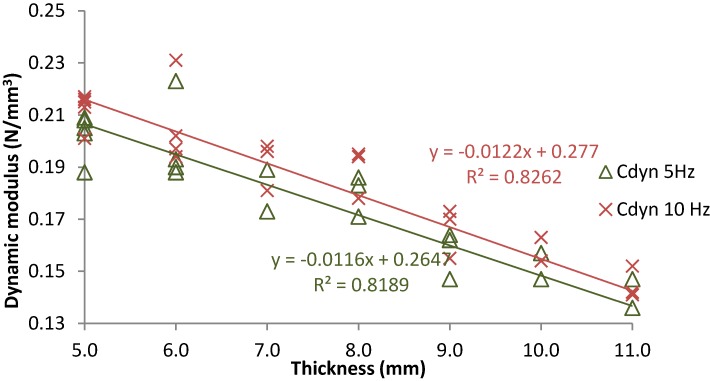
Dynamic results recorded from tire USP with different thicknesses.

Figure 6 shows the dynamic stiffness modulus (at 5 Hz and 10 Hz) recorded for tire USP with thickness between 5.0 mm and 11.0 mm. Results fit to a lineal law in reference to element thickness for both load frequencies, obtaining a wide range of elastic solutions capable of being used in transition zones to achieve a smooth variation of vertical stiffness. Regarding the effect of load frequency, on the one hand the increase of this parameter causes the material stiffening; on the other hand, when dynamic and static results are compared a ratio close to 1.5–2.0 is obtained. Thus, it is understood that load frequencies also have an important effect on the study of tire elements’ behavior.

#### 3.1.3. Under Ballast Mats

**Figure 7:**
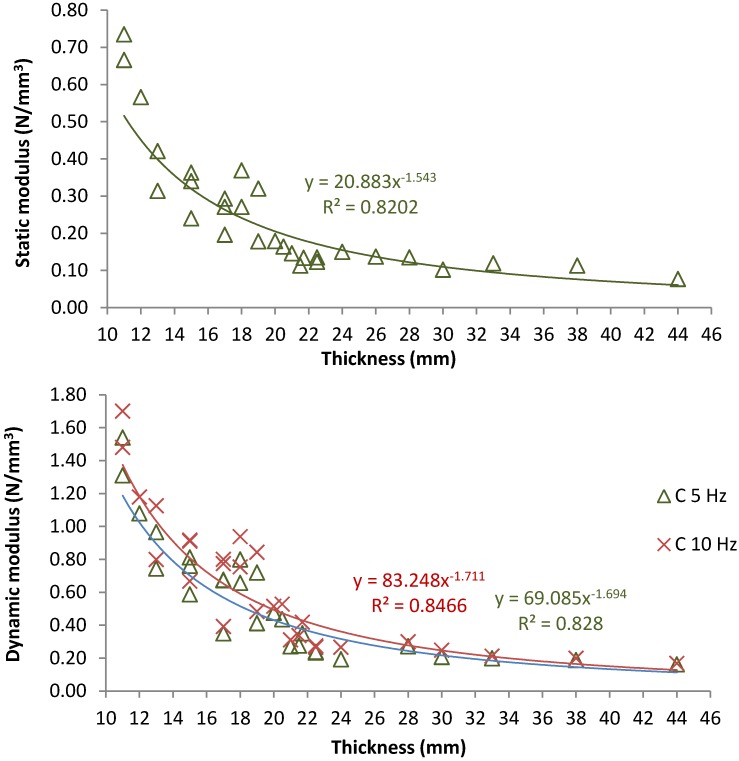
Effect of thickness on tire mats mechanical performance, static and dynamic results.

Figure 7 shows the static and dynamic (at 5 Hz and 10 Hz) results recorded for tire mats with diverse thickness values. This study was developed according to the DBS 918 071-01 which indicate the use of flat plates to load mats during the tests. Results demonstrate that it could be adequate to increase tire mats thickness to obtain more flexible elements, which are apt to be used in railway tracks such as those used on a high-speed railway.

Thus, according to DBS 918 071-01 specifications, thickness among 19.0 and 38.0 could be used for tire UBM in conventional railway tracks (where trains speed is lower than 230 km/h), whereas the thickness must be higher than 21.0 mm for high speed tracks since the static modulus should be close to 0.10–0.15 N/mm^2^. According to standard elastic mats [32], the most appropriate thickness values are between 15–30 mm, although 40 mm could be possible in some railway lines. Nonetheless, tire mats presented slightly higher static and dynamic stiffness in comparison with the results recorded for other author for UBM with 35 mm of thickness and made of crumb rubber and resin [8]. Once again, the effect of the load frequency plays a significant role since higher stiffness is presented when this parameter is increased.

### 3.2. Resistance Capacity of Tire Elements

#### 3.2.1. Rail Pads

With the aim of determining the durability of rail tire pads, a fatigue test applying inclined (33°) dynamic loads over a piece of rail settled on a sleeper which incorporated a soft fastening system. This configuration (without ballast) tried to simulate only the contact rail-pad-sleeper when the passage of trains. Table 3 shows the rail movement variations (for the maximum force, 83 kN; and minimum force, 5 kN) after fatigue (3 × 10^6^ load cycles) in relation to those recorded before this process. It was observed that the greatest changes in rail movements occur in the railhead, which could be due to the fact that railhead movements are higher than those measured in the foot, which is associated to the use of a flexible fastening system. Nonetheless, movements values recorded in both cases (in railhead and in the foot) show limited variations in tire pads behavior. Thus, these materials could be appropriate for their use in railway tracks.

**Table 3:** Variation of rail movements due to fatigue process.

Rail movements	D_1_ railhead	D_2_ railhead	D_1_ foot	D_2_ foot	D_3_ foot	D_4_ foot
*for F_max_*	0.299	0.258	0.072	0.052	0.024	0.033
	0.279		0.049			
*for F_min_*	0.009	0.063	0.020	0.013	0.046	0.026
	0.036		0.026			

In addition, in order to analyze in-depth the fatigue resistance of tire pads, the variation in material static and dynamic stiffness was studied. Results indicated that the static stiffness of tire pads increased in 16.38% as well as 13.10% in the case of dynamic stiffness, obtaining in both cases a material stiffening lower than 25.0%, which proves the ability of using tire pads in railways tracks [25]. Besides, the change in material capacity to dissipate energy was studied; recording values lower than 28% and 14% in static and dynamic tests, respectively.

**Table 4:** Change in tire pads mechanical properties due to climatic actions. Bold italics show the variation between the properties before and after applying climatic processes.

Parameter		Hot-cold cycles	Thermal ageing	Freeze-thaw cycles
static stiffness 20–95 kN (kN/mm)	before	94.01	96.94	88.93
	after	101.43	104.6	93.39
	***variation* (%)**	***7.29***	***7.15***	***4.46***
static stiffness 100–200 kN (kN/mm)	before	263.91	257.27	276.03
	after	265.80	266.82	260.24
	***variation* (%)**	***0.52***	***3.60***	***−5.36***
Dynamic stiffness (kN/mm)	Before	334.82	305.13	354.65
	After	380.50	337.84	420.97
	***variation* (%)**	***11.94***	***10.12***	***23.61***

Table 4 represents the effect of 3 artificial climatic processes (thermal ageing, hot-cold and freeze-thaw) on the static (between 20–95 kN and 100–200 kN) and dynamic (at 4 Hz between 20–95 kN) response of tire pads. The stiffness variations obtained were lower than 20% (with exception of dynamic stiffness under the process of freeze-thaw), which indicates an adequate resistance of the material to climatic agents.

#### 3.2.2. Under Sleeper Pads

**Figure 8:**
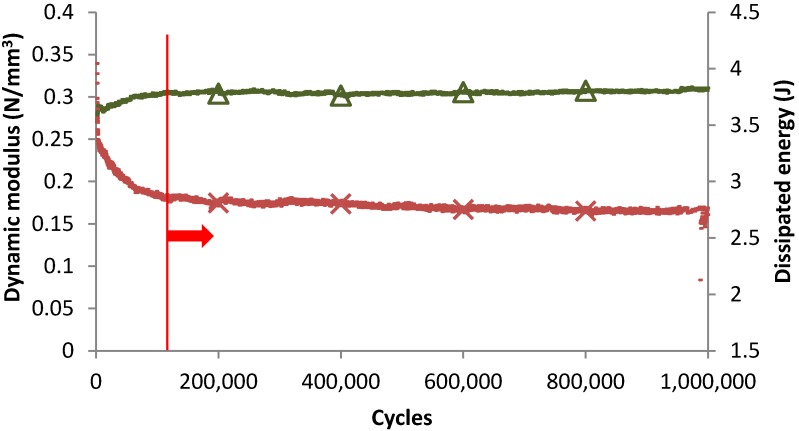
Evolution of dynamic modulus and dissipated energy values during tire USP fatigue process.

In order to determinate the ability of tire USP for their use in railway tracks, the resistance to mechanical fatigue was studied. Figure 8 shows the evolution of the dynamic modulus and dissipated energy values recorded in a fatigue test developed in a ballast box where the load was applied over a sleeper piece incorporating a tire pad. Results prove that the stiffness modulus increased during the first load cycles, which could be associated with ballast compaction. Nonetheless, the modulus stiffness and capacity to dissipate energy were stable from 100,000 cycles to 1,000,000 (total number of loading cycles), which shows a good long-term behavior of the tire USP.

The static and dynamic stiffness of tire under sleeper pads before and after the fatigue process were also evaluated, in order to determine the material durability. It was seen that long-term dynamic loads provoke the increase in tire USP stiffness, being this changes lower than 15.00%. Thus, under sleeper pads from used tires could be appropriate to modify railway tracks without increasing maintenance tasks due to the deterioration of elastic elements.

Table 5 presents the tensile strength (σ) and elongation (ε) capacity variation as a result of developing three different atmospheric processes (thermal ageing, hot-cold and freeze-thaw cycles). Positive values mean that the strength and deformation capacity of the material is increased. Tire USP presents good resistance to climatic deterioration since the strength and elongation variations are positives, with exception of the samples under thermal ageing which recorded lower capacity to deform as a consequence of the heating process. Thus, tire USP have appropriate resistance to be used as elastic components in railway tracks.

**Table 5:** Variation of tire USP properties after climatic processes.

Original tire USP properties		Variation of the material properties					
		Thermal ageing		Hot-cold cycles		Freeze-thaw cycles	
σ (MPa)	ε (%)	σ (%)	ε (%)	σ (%)	ε (%)	σ (%)	ε (%)
24.7	22.3	8.5	−39.5	1.2	2.7	33.8	20.6

#### 3.2.3. Under Ballast Mats

Table 6 shows the changes in static and dynamic stiffness modulus of tire mats after applying 3 artificial processes that reproduce the climatic actions (heat, frost and long-term water action) expected during their service life. Results indicate that water action provokes the reduction of the material stiffness whereas thermal ageing leads to higher stiffness modulus. This is more important when the load frequency is increased. Regarding freeze-thaw process, there is no clear trend, although all values are lower 20%. Based on results, tire UBM presents adequate values of resistance to climatic deterioration, which makes them apt to be used in railway tracks.

**Table 6:** Results of tire mats resistance to climatic deterioration. Bold italics show the variation between the properties before and after applying climatic processes.

Parameter		Long-term water	Thermal ageing	Freeze-thaw cycles
C_stat_ (N/mm^3^)	before	0.125	0.134	0.113
	after	0.123	0.151	0.130
	***variation* (%)**	***−1.62***	***12.69***	***15.04***
C_dyn 5_ _Hz _(N/mm^3^)	Before	0.238	0.292	0.276
	After	0.232	0.351	0.290
	***variation* (%)**	***−2.52***	***16.81***	***5.07***
C_dyn 10_ _Hz _(N/mm^3^)	Before	0.294	0.309	0.337
	After	0.275	0.418	0.312
	***variation* (%)**	***−6.91***	***26.07***	***−7.41***

### 3.3. Study of the Effect of Tire Elements in a Ballast Box

**Figure 9:**
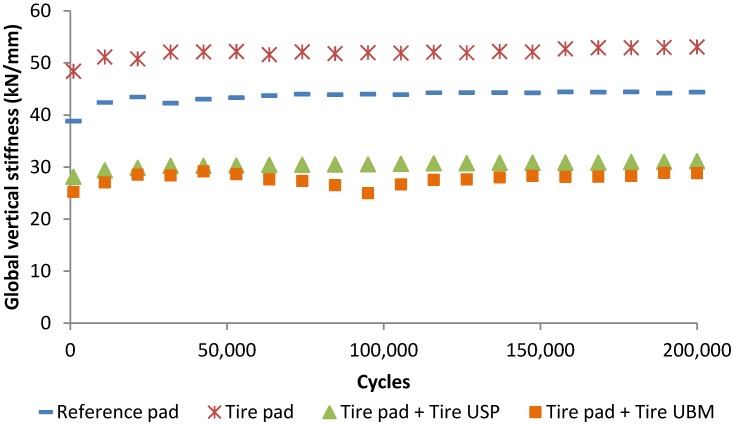
Evolution of vertical stiffness of a ballast box with different tire elastic solutions.

**Figure 10:**
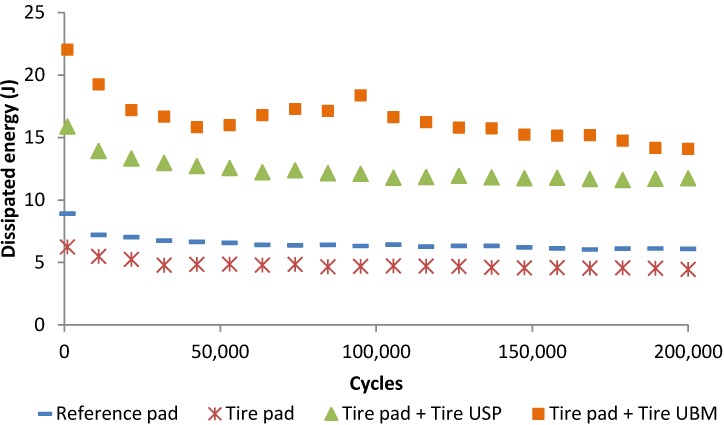
Effect of tire elements in the capacity of the ballasted system to dissipate energy.

A ballast box study was carried out by using different elastic solutions with the aim of evaluating the effect of tire elastic elements in more realistic conditions. Nonetheless, to determine the optimal solution to be used in real railway tracks would be necessary to carry out a more in-depth study. Figure 9 and Figure 10 display the evolution of dynamic vertical stiffness and dissipated energy values (respectively) for the different cases studied. It is seen that the use of tire pads led to a slight increase in the dynamic stiffness of the ballast box configuration as well as the dissipated energy values were decreased in comparison to the values recorded for a reference commercial pad. This fact could be related to the difference of the dynamic stiffness between tire pads (near 350 kN/mm for a thickness close to 7.0 mm) and the reference pad (roughly 220 kN/mm).

When tire USP and UBM are used, the stiffness of the ballast box decreases remarkably whereas the dissipated energy values increase since these elements present low stiffness values. Nevertheless, it is important to considerer that the use of UBM led to a slight change in the evolution of stiffness and dissipated energy values, which could be related to the decompaction of the ballast layer as a result of wide deflections and vibrations of the ballast box when mats are used. This could be influenced by the flexibility of the system employed in the laboratory tests. Thus, a study in a real scale section could be necessary to analyze the effect of tire mats.

**Figure 11:**
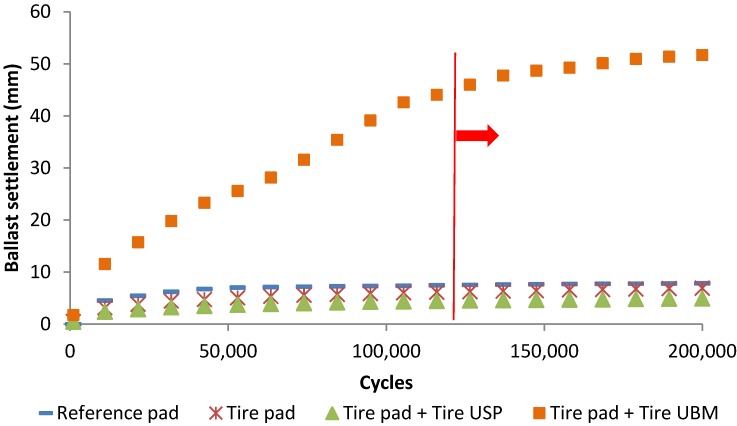
Evolution of the ballast settlement depending on the tire elements used.

On the other hand, Figure 11 shows the effect of elastic elements in ballast layer settlement. It is observed that the use of tire rail pad reduces the system settlement in comparison with the reference rail pad, being also positive the incorporation of tire USP to reduce ballast settlements as a consequence of the decrease of the stress transmitted to the ballast layer from the sleepers. However, the incorporation of tire UBM caused an important increase in settlement as a result of the decrease of stiffness and increase of dynamic movements. Nonetheless, after 120,000 load cycles, the increase in ballast settlement was significantly reduced, which means that the long-term behavior of the system could be appropriate to reduce track deterioration.

## 4. Conclusions

The aim of the research carried out was to determinate the viability of developing elements from deconstructed end-of-life tires to be used as elastic components in railway infrastructures, in order to improve track mechanical performance as well as reducing landfill disposal. Laboratory tests were performed to measure the influence of tire thickness in the behavior of rail pads, USP and UBM as well as the materials resistance capacity and their effect on ballasted tracks performance. Based on the results obtained the following conclusions can be derived:

- Due to the decrease in vertical static and dynamic stiffness of elastic elements when its thickness is increased, a wide range of wide range of rail pads with diverse mechanical response can be used in railway tracks. In addition, the dynamic stiffness of tire pads is comparable to that presented by standard rail pads (although it depends on the material of the commercial pads).
- According to other experiences, the most adequate thickness values for the use of tire rail pads in high speed railway are in the range of 7.5 to 9.0 mm (soft pads) whereas tire pads with thickness lower than 7.5 mm could be used in tracks that require stiffer pads.
- Although the thickness of USP from used tires is limited since the outer layer of this waste is usually between 4.0–11.5 mm (whereas standards USP thickness is up to 20 mm), these materials present appropriate stiffness values to be used as soft elastic elements in railway tracks.
- As standard UBM thickness is up to 30–40 mm, it is necessary to join diverse tire layers to obtain elastic materials with appropriate mechanical performance to be used as components on the railway. According to the standard employed in this study, tire UBM thicknesses higher than 21.0 mm are required to obtain flexible elements for their use in lines with speeds greater than 230 km/h.
- Tire elements presented adequate resistance to mechanical fatigue and climatic deterioration and therefore could be apt to be used as railway components.
- The use of tire elements allowed varying the mechanical response (their use could reduce settlement and vertical stiffness as well as higher dissipated energy values may be obtained) of a ballast box used to analyze the effect of these components. Nevertheless, a more in-depth study with real scale testing would be advisable to prove the effectiveness of the tire elements at the same time that the optimal design parameters are defined.

Based on the results recorded in this work, it has been provided the viability of developing elastic elements to be used in railway infrastructures. This application would allow reducing construction costs are reduced as well as an abundant waste is reused. Nonetheless, a more in-depth study focused on material long-term behavior could be necessary.
